# Contrast-enhanced voiding urosonography - urodynamics in patients with neurogenic bladder dysfunction: a pilot study within pediatric urological practice

**DOI:** 10.3389/fruro.2026.1891758

**Published:** 2026-10-02

**Authors:** Alexandra Wilke, Tina Hörz, Monica Pleul, Carola Hörz, Katrin Schuchardt, Guido Fitze, Christian Kruppa

**Affiliations:** Department of Pediatric Surgery, Faculty of Medicine and University Hospital Carl Gustav Carus, Dresden University of Technology, Dresden, Germany

**Keywords:** contrast-enhanced voiding uronosonography, neurogenic, urinary bladder, urodynamics, vesico-ureteral reflux

## Abstract

**Background:**

The progressive loss of kidney function is a major cause of morbidity and mortality in children with neurogenic bladder dysfunction. A consequent follow-up, including repeated urodynamics combined with detection of vesicoureteral reflux (VUR) is crucial for the individual treatment of the urinary tract. The aim of our study is to investigate whether contrast-enhanced voiding urosonography -urodynamics (ceVUS-UD) can be used in routine urological diagnostics. Due to its radiation-free nature in a more comfortable environment, this would allow the examination to be performed frequently enabling more accurate monitoring of bladder function.

**Methods:**

Since January 2025, 19 patients with neurogenic bladder dysfunction have undergone simultaneous ceVUS as part of urodynamic follow-up. The children were 11.4 years old on average (9 male, 10 female) and showed one of the following underlying conditions: spina bifida, detrusor hyperactivity after spinal cord injury, caudal regression syndrome, epispadias, infantile cerebral palsy or myotonic dystrophy type 1. Two-thirds of the patients performed self-intermittent catheterization.

**Results:**

CeVUS-UD can be safely performed as part of routine urodynamic testing without interfering with urological measurements. Previously unknown secondary VUR (sVUR) was detected in six patients (31.6%). In one case, a significant decrease of vesical pressure during the occurrence of reflux was observed. For five children, this led to a change in treatment .

**Conclusions:**

In this study, CeVUS-UD provides a feasible, safe and radiation-free tool for characterizing neurogenic bladder dysfunction. Whether ceVUS-UD can serve as an alternative to video urodynamics (VUD), and whether routine monitoring of sVUR translates into improved long-term kidney outcomes, remains to be established in further studies.

## 1 Introduction

The progressive loss of kidney function is a major cause of morbidity and mortality in children with neurogenic bladder dysfunction. Reduced bladder compliance, increased intravesical pressure and urinary retention are important risk factors for recurrent urinary tract infections, secondary vesicoureteral reflux (sVUR) and hydronephrosis potentially leading to renal failure. Early intervention based on urodynamic findings is crucial for improving the long-term preservation of kidney function and quality of life. Refractory cases particularly benefit from close urodynamic monitoring, allowing the therapy to be adjusted (1, 2).

In cases of spinal cord malformations, systematic reviews and expert consensus recommend early and regular urodynamics (UD): the first examination during hospitalization after birth or within the next 3 months, followed by every 6 months during infancy, and annually during early childhood. This forms the basis for early adequate treatment, such as intermittent catheterization and anticholinergic therapy, thereby leading to improved long-term renal outcomes (1–3). Similarly, all other children suspected of having a neurogenic bladder due to various causes like tethered cord, inflammation, tumors, trauma, or children with anorectal malformations, should undergo urodynamic testing and begin appropriate therapy without delay (3).

In conventional UD without simultaneous imaging, sVUR may be overlooked as an overestimation of bladder compliance and delay the necessary initiation of appropriate therapy (4). Only when all causes of artifacts are critically considered, it is possible to maintain the quality of UD and its interpretation (5).

Therefore, various guidelines recommend video urodynamics (VUD) as the optimal examination method for patients with neurogenic bladder dysfunction (6, 7), which involves X-ray exposure and is usually performed under less comfortable conditions. An alternative imaging is the contrast-enhanced voiding ultrasonography (ceVUS) applying SonoVue^®^, which was approved by the Food and Drug Administration in 2016. It is a more modern method for evaluating VUR and other urogenital abnormalities in a more patient-friendly setting (8, 9). CeVUS can be performed as a video or image recording at various stages of bladder filling and during micturition without any radiation exposure or interfering artifacts, leading to a reduced person’s lifetime radiation exposure (8, 10). Furthermore, studies proved that ceVUS has equivalent or higher sensitivity than voiding cystourethrography (VCUG) in detecting VUR and intrarenal reflux (11).

This study investigates whether the combination of UD and ceVUS to detect sVUR can be performed safely and effectively in a comfortable environment. Aiming to share experience of this emerging technique with to date limited published data (12, 13). We hypothesize that excellent imaging combined with adequate measurements may enable better-informed decision-making regarding further therapy; whether this translates into prevention of future loss of kidney function remains to be determined. 

## 2 Materials and methods

### 2.1 Study design, data sources, statistical methods

Between January 2025 and November 2025, 19 patients with neurogenic bladder dysfunction have undergone simultaneous ceVUS as part of UD follow-up. Written informed consent was obtained from the parents of all patients. Demographic data such as gender, diagnosis, and age at the time of examination were recorded prospectively, additionally to the examination outcomes. The age-dependent adequate bladder capacity in ml was calculated using the following formula: (age+1) *30 to a maximum of 400 ml. Patient data was analysed using SPSS, applying descriptive statistics. This study was designed as a feasibility investigation. No direct comparison with a reference standard (VUD) was performed.

### 2.2 Patients

The children were 11.4 years old on average (9 male, 10 female) and showed one of the following underlying conditions: spina bifida, detrusor hyperactivity after posttraumatic or iatrogenic spinal cord injury, caudal regression syndrome, epispadias, infantile cerebral palsy or myotonic dystrophy type 1 (**Table 1**).

**Table 1:** Patients.

Patients	19
Mean age (years)	11.4 (± 4.6)
Female/Male	10/9
Congenital	16
Posttraumatic, postsurgical	3
Neurogenic bladder	17
Non-neurogenic neurogenic bladder	2
Spina bifida	6
Myelomeningocele (MMC)	5
Caudal regression	6
Infantile cerebral palsy (ICP)	3
Myotonic dystrophy Type 1	1
Epispadias	1
S/p ureteral reimplantation	2
Mitrofanoff stoma	2
Vesicostomy	1

Two-thirds of the patients performed self-intermittent catheterization (SIC). Only 3 children were urinary continent. However, 11 (57.9%) reached social urinary continence under SIC and medical treatment. The vast majority (17, 89.5%) presented fecal continence, with one third of the patients performing irrigation therapy. Despite therapy, one child remained obstipated. 63.2% (n = 12) received one of the following medicaments: oral anticholinergics (Propiverine), oral antispasmodics (Trospium chloride) or intravesical antispasmodics (Oxybutynin). Three children underwent a urinary tract infection (UTI) within the last 12 months. None of the patients had a pyelonephritis in the last 12 months (**Table 2**).

**Table 2:** Current anamnesis.

Patients	19 (100%)
Infection history	
S/p pyelonephritis	9 (47.4%)
S/p urinary tract infection (UTI)	14 (73.7%)
UTI in the last year	3 (15.8%)
Antibiotic prophylaxis	3 (15.8%)
Bladder management	
Self-Intermittent Catheterization (SIC)	13 (68.4%)
Daily frequence if SIC	4.3
S/p urinary retention	3 (15.8%)
Urinary continence (dry day and night)	3 (15.8%)
Social continence (dry for ≥3 hours during the day)	11 (57.9%)
Bowel management	
Fecal irrigation, retrograde (Peristeen^®^)	6 (31.6%)
Fecal continence (no unplanned bowel movements and minimal soiling (<2 smears per week))	17 (89.5%)
Obstipation (despite laxatives)	1 (5.3%)
Pharmacotherapy	
Oral anticholinergics (Propiverine)	7 (36.8%)
Oral antispasmodics (Trospium chloride)	2 (10.5%)
Intravesical antispasmodics (Oxybutynin)	5 (26.3%)

### 2.3 Examination (ceVUS- UD) setting

Contrast-enhanced voiding urosonography (ceVUS) was performed to assess VUR under radiation-free, real-time ultrasound imaging trying to create a children-friendly setting close to their parents (**Figure 1**). All examinations were conducted by a pediatric surgeon using a high-resolution ultrasound system equipped with a convex transducer in a standardized pediatric setting (5MHz). In Germany, it is common for pediatric surgeons to be trained in ultrasound diagnostics and pediatric urology. No radiologist was involved in the examinations. Patients were placed in supine position. Following age-appropriate preparation of urodynamics done by an experienced nurse, transurethral bladder catheterization was carried out under aseptic conditions placing a 6 Fr catheter. After fully emptying the bladder, 1ml of ultrasound contrast agent (SonoVue^®^) was instilled intravesically. Afterwards, the bladder was filled with sterile saline at a pace of 10ml/min until an age-appropriate bladder volume was achieved. If intravesical pressure rose above 20 cmH_2_0, minimal urine overflow occurred, or the patient reported first discomforts, the filling rate was halved to 5 ml/min. The examination was discontinued if the child was unable to tolerate further filling due to discomfort or if continuous leakage occurred. Most children were able to void with the catheter in place. If voiding was not possible, the catheter was removed to allow micturition and simultaneous ceVUS evaluation to detect possible VUR.

**Figure 1:**
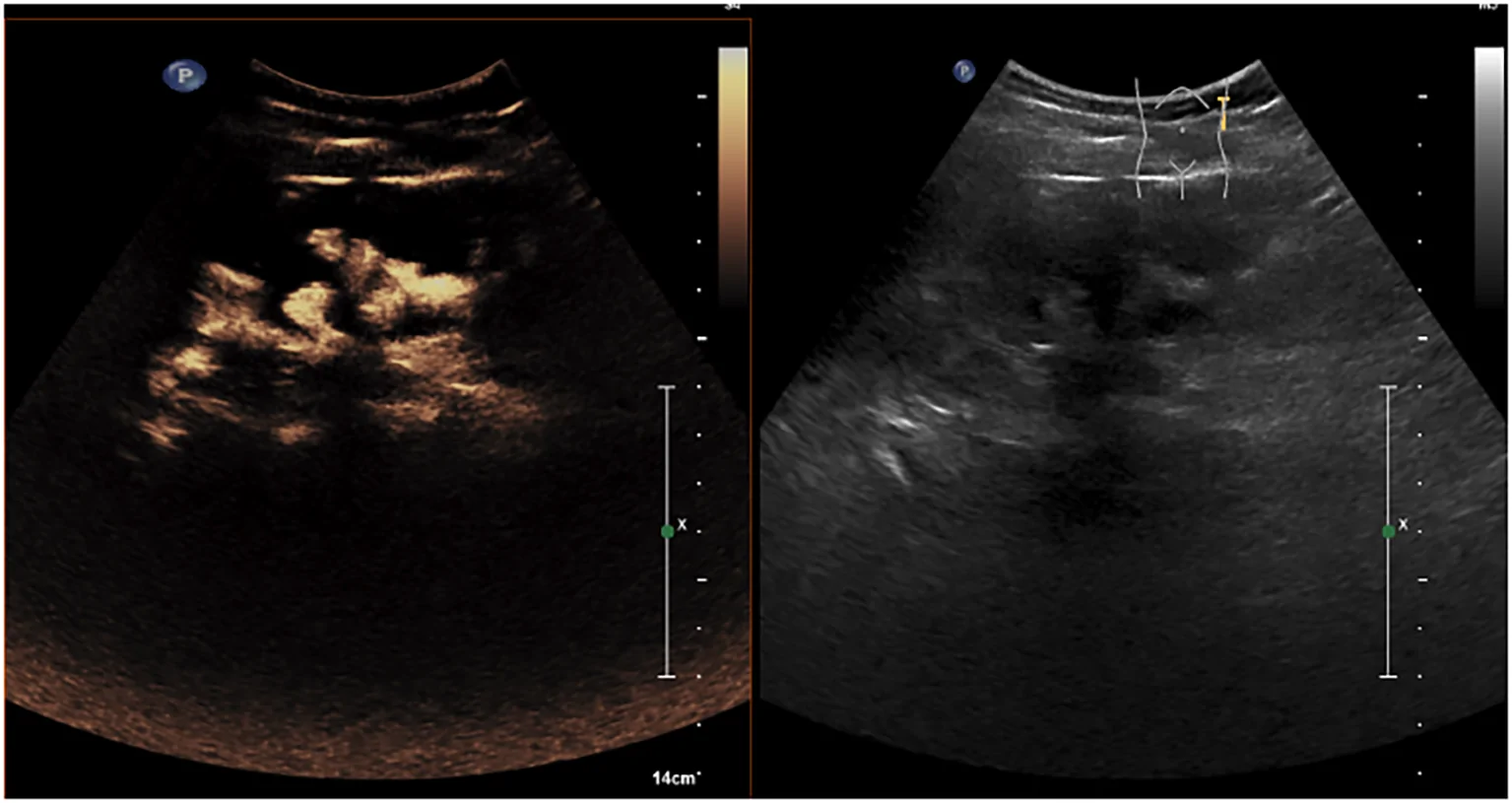
Prepared diagnostic setting of ceVUS before the patient enters. The pediatric urologist sits on the left in front of the ultrasound, the patient will lie down on the examination couch. Parents can take place next to their child. The pediatric nurse monitors the pressure curves of the urodynamics.

Transverse and longitudinal views of the bladder and longitudinal views of the ureters and kidneys during the early, mid and late bladder filling and voiding phases or urinary overflow were obtained by ceVUS. With the examination focused on the detection of sVUR during UD we did not assess the urethra or the bladder neck during the UD. Alterations in intravesical pressure occurring during ultrasonographic examination were noted. A post-voiding assessment of the lower and upper urinary tract was conducted to exclude residual urine or delayed reflux. Despite contractures, scoliosis, or spina bifida in a large proportion of the cohort, sonographic assessment of the bladder, ureters, and kidneys was feasible in all patients. If children were seated during urination, ceVUS could be performed as well detecting sVUR at least a grade II. All children regularly performing SIC received another ultrasound to ensure sufficient catheterization.

## 3 Results

Although the duration of each examination was not recorded, the extent of imaging with ceVUS was limited by the UD itself and was not determined by the imaging time.

The contrast medium initially administered via the bladder catheter was visible throughout the entire examination period. Gentle sonographic examination of the bladder resulted in a transient increase in intravesical pressure of 1–2 cmH_2_O, with an immediate decrease after removing the probe within seconds. Lateral pressure applied with the ultrasound probe to improve visualization of the kidneys did not increase intravesical pressure, even when substantial pressure was applied.

Previously unknown sVUR was detected in six patients (31.6%) (**Figure 2**), observing a significant decrease of vesical pressure during the occurrence of left-sided VUR III° in one case from 37 cmH_2_O to 20 cmH_2_O within 13 seconds (**Figure 3**). Those patients showed no VUR on prior fluoroscopy and had no signs of UTI since then. All instances of VUR occurred at maximal bladder filling while urinary leakage was observed. The pressure at which VUR occurred therefore corresponds to the leak point pressure (LPP) and to maximum bladder capacity. The patient characteristics are presented in detail in **Table 3**.

**Figure 2:**
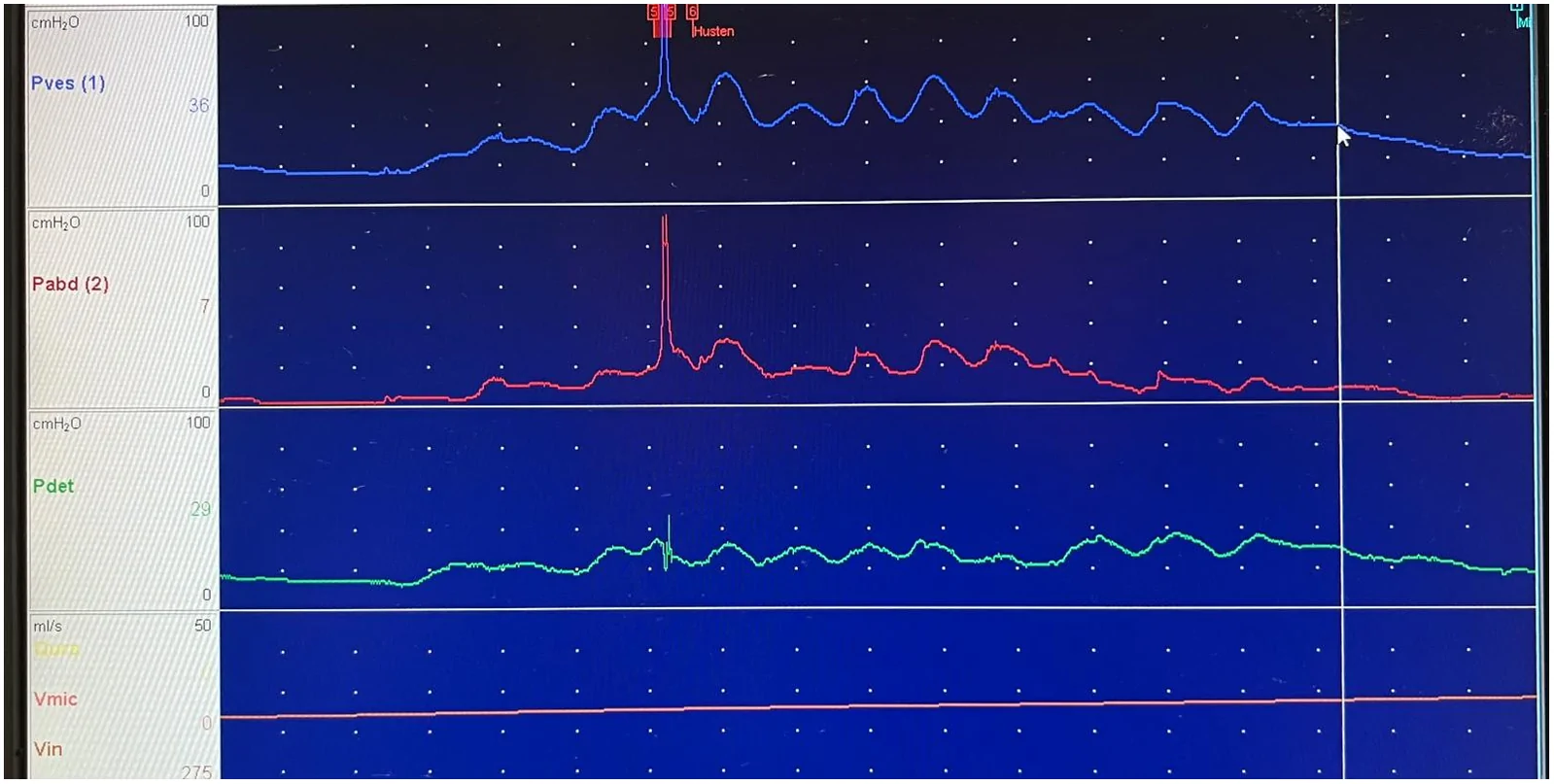
Contrast-enhanced urosonography revealing a secondary VUR IV° into the left kidney at an intravesical pressure of 50 cmH_2_O in patient 3.

**Figure 3:**
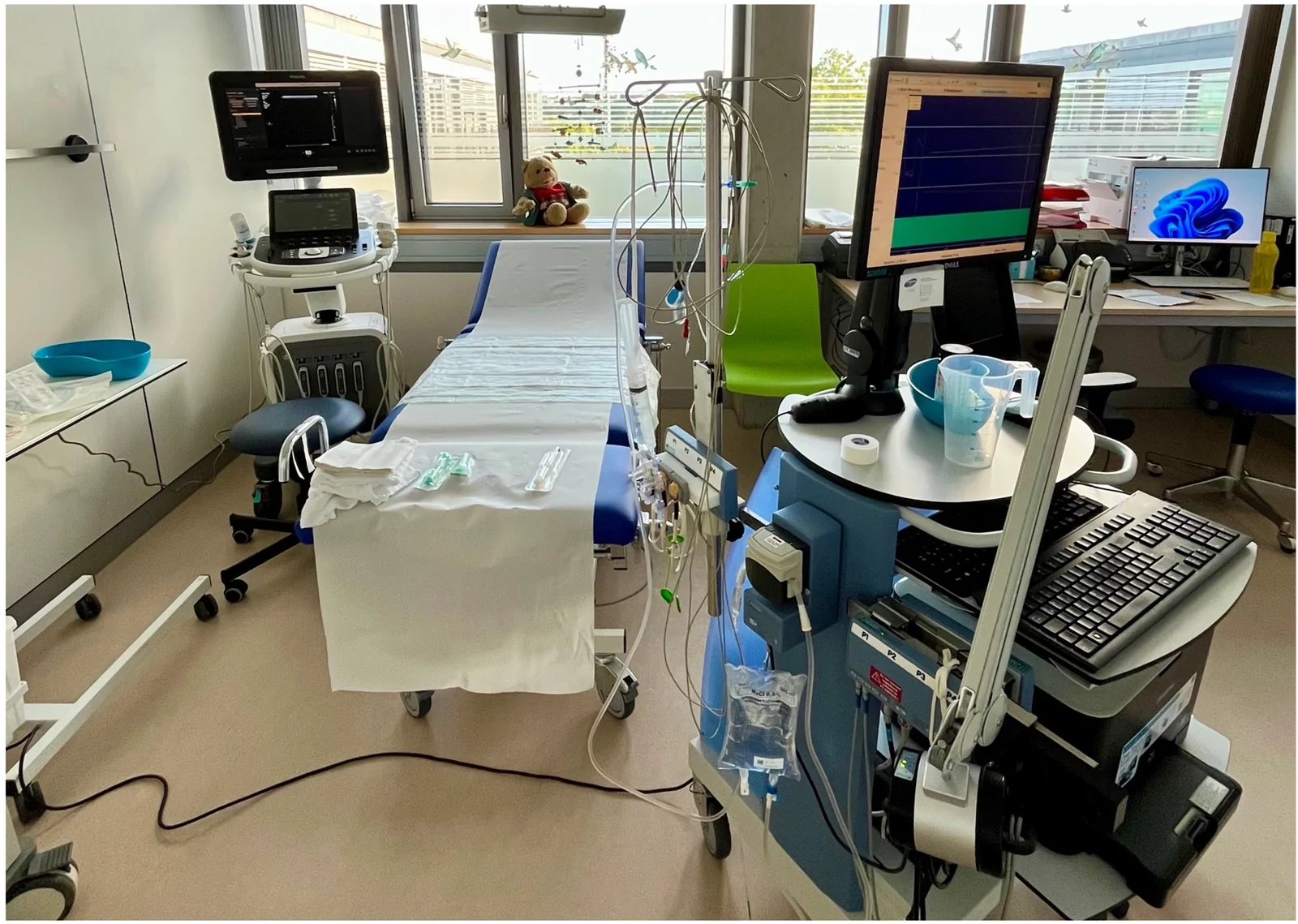
Urodynamic curve showing the significant decrease of vesical pressure during the occurrence of secondary VUR III° into the left kidney from 36 cmH_2_O (pointer) to 20 cmH_2_O within 13 seconds. The red mark indicates the moment when the patient coughed, which was used to assess appropriate transmission of bladder and abdominal pressures.

**Table 3:** Patients with secondary VUR.

	Patient 1	Patient 2	Patient 3	Patient 4	Patient 5	Patient 6
Age in years	6.4	13.5	8	5.3	7.4	14.5
Female/Male	F	F	F	M	F	M
Diagnosis	Caudal regression	Epispadias	Spina bifida	Spina bifida	Spina bifida, MMC, Arnold-Chiari-malformation, tethered chord	Cervical MMC
S/p urological surgery	–	Mitrofanoff	Vesicostomy	–	–	–
Urinary continence	–	–	–	–	–	+
Fecal continence	+	+	+	+	+	+
Obstipation	–	–	–	–	–	–
Febrile UTI since last urodynamics	–	–	–	–	–	–
Medication	Oral Propiverine	–	Oral Propiverine	–	–	Oral Propiverine
SIC	–	2x/d	5x/d	–	–	2x/d
Max. bladder capacity (ml), percentage of calculated adequate bladder capacity	132 (59.5%)	150 (37.5%)	60 (22.2%)	80 (42.4%)	250 (99.2%)	280 (70.0%)
Urine leakage	+	+	+	+	+	+
Overall P_ves_	30	10	20	20	20	22
LPP_ves_ (cmH_2_0)	45	12	50	60	40	40
Compliance (ml/cmH_2_0)	2.9	12.5	1.2	1.3	6.25	7
Residual urine	–	+, (SIC inefficient)	+, (SIC inefficient)	+, - (SIC)	+	+, - (SIC)
Sensitivity	+	+	+	+	+	+
VUR° left; right (all after leakage)	3; 0	3; 4	4; 0	1; 0	2; 2	3; 0
Therapy change, step 1	- MAG-3 planned	SIC-training, intravesical Oxybutynin	SIC-training, intravesical Oxybutynin	Propiverine	MAG-3 planned, Propiverine, SIC 4x/d	Antibiotic prophylaxis, Propiverine, increased frequency in SIC (3x/d)

## 4 Discussion

The study aimed to examine feasibility of ceVUS-UD in all its facets. In this context, the environment, feasibility, and the equivalent diagnostic value regarding the detection of pathologies play a key role. Within this scope, the detection of vesicoureteral reflux (VUR) plays a central role, as the principal methodological difference between ceVUS-UD and conventional video-urodynamics (VUD) lies precisely in the modality used to detect VUR — ultrasound-based contrast detection versus fluoroscopic imaging. It is therefore this aspect of the two techniques that differs most substantially, and consequently most relevant to compare when evaluating feasibility and diagnostic equivalence. Furthermore, VUR is of central importance for the long-term prognosis of kidney function. We therefore placed particular emphasis on VUR and its detection, since one of the questions motivating this study was whether the combined use of ceVUS-UD can improve the diagnostic yield and characterization of VUR in its various forms and grades, compared with conventional methods.

Contrast-enhanced voiding urosonography (ceVUS), has become established in pediatric radiology as a sensitive, radiation-free technique for detecting VUR and assessing lower urinary tract function. Its increasing clinical adoption aligns with the broader shift toward minimally invasive, low-morbidity diagnostic strategies in pediatric urology, aiming to reduce radiation exposure and procedural burden while maintaining or improving diagnostic accuracy. In many centers, ceVUS has become an integral part of standardized imaging algorithms for children with suspected or known VUR and is increasingly used as an alternative to fluoroscopic studies, particularly in follow-up settings (14).

It must be acknowledged that, in order to avoid distortion of intravesical pressure, the lower urinary tract was not assessed in detail in this study. However, Kennedy et al. demonstrated that changes of the bladder neck during ceVUS- UD and visualization of the urethral anatomy can be achieved without compromising urodynamic quality (12). A gentle ultrasound examination of the bladder as part of the ceVUS-UD resulted in a temporary increase in intravesical pressure of 1–2 cmH_2_O, which settled within seconds immediately after the probe was removed and therefore probe-related pressure relieved. Although this study did not focus on detrusor overactivity, SonoVue^®^ has not been reported to increase its occurrence in prior studies (15).

In the present cohort, which consisted of non-selective consecutive children, the detection rate of sVUR was 31.6% despite the absence of clinically apparent UTIs within the past year.

For five children, the detection of sVUR through ceVUS-UD lead to the treatment adjustments . Urodynamic characteristics of children with detected VUR were further analyzed. This study did not further investigate on the non-refluxive cohort.

Analysis of the evaluated patient cohort indicates that neither adequate bladder capacity (patient 5), low leak point pressure (patient 2), oral anticholinergic therapy (patients 1, 3, and 6), nor the absence of constipation under appropriate management (all patients with VUR) were protective against the occurrence of sVUR. No urinary tract infections were recognized in our cohort since the last examination that could have served as a clinical marker to prompt indication for sVUR-diagnostics like VUD or ceVUS-UD.

Early identification of sVUR, especially silent or secondary forms, through more frequently performed ceVUS- UD may contribute to preventing secondary renal damage by enabling timely therapeutic adjustments (14). In this patient population, the findings also support consideration of a greater intensification of conservative management, including self-intermittent catheterization, anticholinergic therapy, and, where indicated, further diagnostic evaluation such as renal scintigraphy to assess the need for surgical intervention like ureteral reimplantation or bladder augmentation.

However, the more comprehensive detection of asymptomatic sVUR in children with neurogenic lower urinary tract dysfunction also raises the question of how invasive subsequent surgical management should be. It remains debatable whether closer surveillance and intensified follow-up could represent a viable alternative to early surgical intervention in selected cases. To adequately address this issue, long-term prospective studies are required.

In clinical decision-making, the risk of progressive renal function impairment associated with conservative therapy or watchful waiting depending on the grade and duration of VUR must be weighed against invasiveness of surgery and anesthesia risk in this complex patient cohort (14). In children with neurogenic bladder dysfunction, endoscopic injection therapy shows resolution rates of 56.7% after a single intervention and 61.7% after two interventions, which are markedly inferior to the 87.8% resolution rate reported for open ureteral reimplantation (16). In contrast, the success rate of open ureteroneocystostomy reaches up to 99% according to the American Urological Association (AUA) guidelines (17).

The findings in patient 6 illustrate an important pathophysiological phenomenon: sVUR can result in a redistribution of intravesical pressure, leading to increased stress on the kidneys even in the absence of infection. These observations support existing hypotheses suggesting that chronic renal injury in VUR may arise not only from recurrent UTIs but also from mechanical and pressure-mediated mechanisms within the urinary tract. This aligns with previous reports that elevated bladder pressures, abnormal voiding dynamics, and VUR itself are significant risk factors for renal scarring and progressive renal dysfunction in children with neuropathic bladder disorders. Children with neurogenic bladder dysfunction appear particularly vulnerable. They frequently present with asymptomatic bacteriuria, often involving multidrug-resistant organisms, which constitutes an additional risk factor for renal deterioration (1, 18, 19).

A distortion of intravesical pressure due to sVUR in UD may lead to an underestimation of renal risk due to apparently normal intravesical pressure during the voiding phase. This again leads to deferring necessary intensification of therapy to protect the renal function.

The usage of ceVUS can reduce the need for VCUG, thereby lowering cumulative radiation exposure (up to 10mGy per examination comparable to a CT-dose (20)) and improving the safety profile of diagnostic follow- up (16). Current German guidelines recommend VUD at 3, 6, and 12 months of age, followed by annual to biennial follow-up examinations. However, due to limited availability and concerns regarding radiation exposure, this schedule does not fully reflect clinical reality. In this context, ceVUS-UD may serve at least as a tool for follow-up assessment after an initial VUD (21). Due to the limited scientific evidence available for this relatively new technique, the authors recommend performing VUD or VCUG as the initial investigation to visualize the lower urinary tract. With increasing experience and further validation, this may no longer be necessary in the future.

CeVUS offers several advantages over VCUG, most notably the ability to perform real-time dynamic assessment of the urinary tract without ionizing radiation. When combined with UD, it enables a comprehensive evaluation of both structural and functional aspects of the lower urinary tract. This multimodal approach is particularly valuable in children with neurogenic bladder dysfunction, for whom individualized therapeutic strategies and continuous monitoring are essential. As such, ceVUS- UD represents a promising radiation-free alternative to VUD, the current standard and therefore totally eliminates the radiation-associated restriction of usage within a standard urodynamics follow-up (6, 16, 22).

Several studies confirm the observation of higher comfort of ceVUS- UD compared to VUD (12, 23). This may be consequence of the less intimidating character of an ultrasound probe compared to the VCUG setting. Children generally did not report feeling affected by the ultrasound examination and, if anything, experienced greater interaction owing to the closer proximity. Also, parents can stay aside their children throughout the whole examination (**Figure 1**).

CeVUS carries a risk of missing first grade VUR compared to VCUG, whereas high-grade VUR is more likely to be underestimated in fluoroscopy (11, 24). In the present study, one patient with VUR I° was detected, demonstrating that low-grade reflux can be visualized even when ceVUS is combined with UD. Furthermore, a recent study comparing ceVUS-UD with VUD reported similar VUR detection rates in both groups, suggesting a reliable sensitivity of this technique although further studies are necessary for confirmation (12). The variability of compliance ranging from 1.2 to 12.5 ml/cmH_2_O confirm the feasibility of ceVUS-UD in very low compliant bladders.

Despite its benefits, ceVUS remains largely confined to specialized centers with appropriately trained personnel and ready access to ultrasound contrast agents. SonoVue^®^ is the currently FDA-approved contrast agent available in Europe. However, implementation challenges persist in institutions where voiding urosonography protocols have not yet been standardized. Nevertheless, broader integration of ceVUS into routine surveillance could lead to earlier detection of clinically silent sVUR, entailing therapy; whether this translates into reduced bladder-related renal stress, delayed onset of renal failure, or improved long-term quality of life remains to be demonstrated in studies incorporating renal functional and long-term outcome data. 

Due to its single center retrospective character, small sample size, lack of control cohort and a short-term follow up, this study faces limitations. The long-term impact of a constant application of ceVUS, particularly regarding renal preservation, prevention of end-stage renal disease or reduction of morbidity and mortality due to earlier recognition of sVUR cannot yet be conclusively determined and only be interpreted as a hypothesis. Larger prospective multicenter studies with standardized ceVUS- UD protocols and comparison to VUD are needed to validate these preliminary results and to better define the role of ceVUS in long-term management strategies for this high-risk population.

As this novel approach seems to offer immense benefits, especially to children with neurogenic bladder disorder, sharing of preliminary results appears to be acceptable at this time of experience.

Further research is warranted to establish evidence-based recommendations and to clarify the long-term impact of ceVUS-UD-guided surveillance on renal outcomes and overall patient well-being.

## 5 Conclusions

CeVUS- UD provides a comprehensive and radiation-free tool for characterizing neurogenic bladder dysfunction while monitoring its progression and treatment. The continuous tracking of sVUR during urodynamics made possible by ceVUS may help to improve the prognosis for kidney function in these patients in the future.

Further studies involving larger patient groups could lead to more accurate diagnostic recommendations for children with neurogenic bladder disorders, including ceVUS.

## Data Availability

The raw data supporting the conclusions of this article will be made available by the authors on request, without undue reservation.
